# Uremic Toxins: An Alarming Danger Concerning the Cardiovascular System

**DOI:** 10.3389/fphys.2021.686249

**Published:** 2021-05-14

**Authors:** Carlos Alexandre Falconi, Carolina Victoria da Cruz Junho, Fernanda Fogaça-Ruiz, Imara Caridad Stable Vernier, Regiane Stafim da Cunha, Andréa Emilia Marques Stinghen, Marcela Sorelli Carneiro-Ramos

**Affiliations:** ^1^Laboratory of Cardiovascular Immunology, Center of Natural and Human Sciences (CCNH), Federal University of ABC, Santo André, Brazil; ^2^Experimental Nephrology Laboratory, Basic Pathology Department, Universidade Federal do Paraná, Curitiba, Brazil

**Keywords:** uremic toxins, cardiorenal syndrome, immune sustem, inflammation, cardiovascular diseases, renal diseases

## Abstract

The kidneys and heart share functions with the common goal of maintaining homeostasis. When kidney injury occurs, many compounds, the so-called “uremic retention solutes” or “uremic toxins,” accumulate in the circulation targeting other tissues. The accumulation of uremic toxins such as *p*-cresyl sulfate, indoxyl sulfate and inorganic phosphate leads to a loss of a substantial number of body functions. Although the concept of uremic toxins is dated to the 1960s, the molecular mechanisms capable of leading to renal and cardiovascular injuries are not yet known. Besides, the greatest toxic effects appear to be induced by compounds that are difficult to remove by dialysis. Considering the close relationship between renal and cardiovascular functions, an understanding of the mechanisms involved in the production, clearance and overall impact of uremic toxins is extremely relevant for the understanding of pathologies of the cardiovascular system. Thus, the present study has as main focus to present an extensive review on the impact of uremic toxins in the cardiovascular system, bringing the *state of the art* on the subject as well as clinical implications related to patient’s therapy affected by chronic kidney disease, which represents high mortality of patients with cardiac comorbidities.

## Uremic Toxins: History, Definition and Classification

Over the past 20 years, interest in understanding uremic syndrome has intensified, especially the impact of uremic toxins on chronic kidney diseases (CKD) and cardiovascular diseases (CVD). Uremic toxins (UT) can be defined as residues of organic compounds which cannot be eliminated from the body, and therefore accumulate in the bloodstream reaching different organs, including the kidneys and heart. Uremic toxins are not fully known but it is well established that they progressively increase in CKD, promoting several functional changes (Vanholder et al., 2003; Glassock, 2008).

The first publication in this area is from 1877 (Mahomed, 1877) and only with the hemodialysis practice, knowledge about uremic toxins became frequent. The concept of UT was gradually built by a small group of researchers from Sweden and France, as biological activities were associated with these molecules. However, studies in this area were subsequently interrupted because their role in uremic syndrome at that time could not be convincingly demonstrated. The topic later resurfaced in the early 1990s by a Belgian nephrology group where they were able to supply new evidence over the biological function attributed to different uremic toxins. In the 2000s, the Vanholder group led the composition of the European Working Group on Uremic Toxins (EUTox), which demonstrated the role of uremic toxins and consolidated this concept around the world (Vanholder et al., 2003).

Uremic syndrome is the result of changes promoted by retention of a myriad of compounds, caused by impaired renal function. The toxicity emanated by the accumulation of these compounds results in the loss of a substantial number of the biological functions of the organism, and its deterioration gradually progresses as the uremic retention becomes more severe due to the progressive loss of renal function (Glassock, 2008; Stinghen et al., 2009; Gryp et al., 2017).

Uremic syndrome must be defined by the presence of uremic toxins in body fluids and their connections with one or more biological and clinical changes (Glassock, 2008). Establishing this connection demands fulfilling a form of Koch’s postulates, as altered by Massry in 1977. Massry/Koch’s requirements for classification of a uremic toxin are shown in Table 1.

**TABLE 1:** Requirements for compounds to be considered uremic toxins (*adapted from* Glassock, 2008).

The toxin must be chemically identified and characterized.
Quantitative analysis of the toxin in biological fluids should be possible.
The level of the toxin in biological fluids must be elevated in uremia.
A relationship between the level of the toxin in biological fluids and one or more of the manifestations of uremia must be present.
A reduction in the level of the toxin in biological fluids must result in the amelioration of the uremic manifestation.
Administration of the toxin to achieve levels similar to those observed in uremia must reproduce the uremic manifestation in otherwise normal animals or man (*in vitro* demonstration of cellular toxicity alone is insufficient to fulfill this criterion).
A plausible patho-biological mechanism should be demonstrated to explain the linkage between the toxin and the uremic manifestation.

Several uremic compounds are identified in serum and plasma samples from CKD patients, and their classification takes place according to their behavior during dialysis (Calaf et al., 2011). In accordance with the European Uremic Toxins Work Group (EUTox) database, there are currently more than 153 uremic solutes listed, and that number should increase over time. Uremic toxins are conventionally divided into 3 groups according to physicochemical characteristics (Vanholder et al., 2003):

(I) Small water-soluble compounds (<500 Da). These molecules include urea and creatinine are easily removed by hemodialysis.
(II) Medium compounds (mainly peptide compounds with molecular weight >500 Da). They have β_2__–_microglobulin (β_2_M) and leptin as prototypes and can only be removed by dialytic membranes which contain large pores.
(III) Protein-bound compounds (including phenols and indoles). These compounds are originated from the metabolism of amino acids of the diet, Whereas removal of protein-bound solutes in a healthy kidney widely depends on tubular secretion, removal by dialysis is limited to the unbound fraction and by convection but is independent of dialyzer pore size (Lesaffer et al., 2000; Meert et al., 2009).

Regarding physicochemical characteristics, the uremic toxins can be classified according to (i) chemical structure: inorganic or organic, (ii) molecular mass/volume: small, medium or large, (iii) distribution in body fluids: hydrophilic [water-soluble], lipophilic, or bound to plasma proteins, ranging from a few nanograms to grams per liter (Vanholder et al., 2003). Table 2 shows the uremic toxins and their respective classifications regarding their physical and chemical characteristics according to the European Uremic Toxins Work Group (EUTox) database. This can be found in the website^1^.

**TABLE 2:** Classification of uremic toxins according physical and chemical characteristics.

Water-soluble (<500 Da) *n* = 44	Middle molecule (>500 Da) *n* = 30	Protein-bound *n* = 31
**Ribonucleoside group**	**Protein group**	**Protein group**
1-methyladenosine (281 Da)	Adiponectin (28,000 Da)	Angiogenin (14,400 Da)
N2,N2-dimethylguanosine (311 Da)	Basic fibroblast growth factor (BFGF) (24,000 Da)	Insulin-like growth factor 1 (IGF-1) (7,650 Da)
N4-acetylcytidine (285 Da)	Complement factor D (26,750 Da)	Leptin (16,000 Da)
Xanthosine (284 Da)	**Peptide group**	Osteocalcin (5800 Da)
1-methylguanosine (297 Da)	Guanylin (1,516 Da)	Retinol binding protein (RBP) (21,200 Da)
Inosine (268 Da)	Uroguanylin (1,668 Da)	Vascular endothelial growth factor (VEGF) (34,250 Da)
1-methylinosine (282 Da)	Vasoactive intestinal peptide (VIP) (3,325 Da)	α1-acid glycoprotein (43,000)
N6-methyladenosine (281 Da)	Adrenomedullin (5,729 Da)	**Hippurate group**
N6-threonylcarbamoyladenosine (378 Da)	Atrial natriuretic peptide (ANP) (3,080 Da)	Hippuric acid (total) (179 Da)
Pseudouridine (244 Da)	Calcitonin gene-related peptide (CGRP) (3,789 Da)	**AGE group**
**Amine group**	Methionine-enkephalin (555 Da)	Pentosidine (378 Da)
*S*-adenosylhomocysteine (384 Da)	Motiline (2,699 Da)	3-deoxyglucosone (162 Da)
Monomethylamine (31 Da)	Cholecystokinin (3,866 Da)	Fructoselysine (308 Da)
Dimethylamine (45 Da)	Clara cell protein (CC16) (15,800 Da)	Glyoxal (58 Da)
Ethylamine (45 Da)	Cystatin C (13,300 Da)	Methylglyoxal (72 Da)
Trimethylamine (59 Da)	Hyaluronic acid (Hyaluronan) (25,000 Da)	N(6)-Carboxymethyllysine (CML) (204 Da)
Trimethylamine-*N*-oxide (75 Da)	Neuropeptide Y (4,272 Da)	**Catecholamine group**
**RCO group**	Resistin (12500 Da)	Dihydroxyphenylalanine (PB-DOPA) (197 Da)
2-heptenal (112 Da)	Vasopressin (ADH) (1,084 Da)	**Amino acid group**
2-hexenal (98 Da)	Endothelin (4,283 Da)	Homocysteine (135 Da)
Nonanal (142 Da)	Degranulation inhibiting Protein I (14,100 Da)	Kinurenine (208 Da)
2-nonenal (140 Da)	Delta-sleep inducing Peptide (848 Da)	Kynurenic acid (189 Da)
2-octenal (126 Da)	Parathyroid hormone (9,225 Da)	**Indole group**
4-decenal (154 Da)	Substance P (1,348 Da)	Indican (295 Da)
Heptanal (114 Da)	β-2-Microglobulin (11,818 Da)	Indole-3-acetic acid (175 Da)
Decanal (156 Da)	β-endorphin (3,465 Da)	Indoxyl sulfate (212 Da)
Hexanal (100 Da)	λ-Ig light chain (25,000 Da)	Indoxyl-β-D-glucoronide (309 Da)
**Amino acid group**	**Cytokine group**	Melatonin (126 Da)
Dimethylglycine (103 Da)	Interleukin-18 (20,000 Da)	Quinolinic acid (167 Da)
4-HO-decenal (170 Da)	Interleukin-1β (32,000 Da)	**Cytokine group**
4-HO-hexenal (114 Da)	Interleukin-6 (24,500 Da)	Interleukin-10 (18,000 Da)
4-HO-nonenal (156 Da)	Tumor necrosis factor alpha (TNF) (26,000 Da)	**Phenol group**
4-HO-octenal (142 Da)		Phenol (94 Da)
**Peptide group**		*p*-Cresylsulfate (187 Da)
β-lipotropin (461 Da)		**Polyamine group**
**Nicotinamide group**		Putrescine (88 Da)
4-pyridone-3-carboxamide-1-β-D-ribonucleoside (272 Da)		**Group (other)**
*N*-methyl-2-pyridone-5-carboxamide (152 Da)		3-carboxy-4-methyl-5-propyl-2-furanpropanoic acid (CMPF) (240 Da)
*N*-methyl-4-pyridone-3-carboxamide (152 Da)		Phenylacetic acid (136 Da)
Nicotinamide (122 Da)		
**Purine group**		
8-hydroxy-2′-deoxyguanosine (283 Da)		
Cytidine (234 Da)		
Hypoxanthine (136 Da)		
Neopterin (253 Da)		
Uric acid (168 Da)		
Xanthine (152 Da)		
**Polyol group**		
Arab(in)itol (152 Da)		
Mannitol (182 Da)		
Erythritol (122 Da)		
Myoinositol (180 Da)		
Sorbitol (182 Da)		
Threitol (122 Da)		
**Guanidine group**		
Argininic acid (175 Da)		
Asymetric dimethylarginine (ADMA) (202 Da)		
Creatine (131 Da)		
Creatinine (113 Da)		
Guanidine (59 Da)		
Guanidinosuccinic acid (175 Da)		
Methylguanidine (73 Da)		
Symmetric dimethylarginine (SDMA) (202 Da)		
Taurocyamine (174 Da)		
α-keto-δ-Guanidinovaleric acid (173 Da)		
α-*N*-acetylarginine (216 Da)		
β-guanidinopropionic acid (131 Da)		
γ-guanidinobutyric acid (145 Da)		
**Aldehyde group**		
Malondialdehyde (MDA) (71 Da)		
**Pyrimidine group**		
Orotic acid (174 Da)		
Orotidine (288 Da)		
Uridine (244 Da)		
**Group (other)**		
Oxalate (90 Da)		
Phenylacetylglutamine (264 Da)		
Urea (60 Da)		

The biological effects promoted by uremic toxins depend on the relationship between synthesis, degradation and elimination, in addition to intracellular distribution and the presence of inhibitors or promoters of the toxin’s action. It was proposed by Glassock (2008) that the toxicity of a substance accumulated in uremia is influenced by the following factors: (1) Rapidity of changes in levels of biological fluids; (2) fluctuations in levels over time (time-averaged versus peak levels); (3) penetration into action sites; (4) intrinsic toxicity versus dependence of metabolism to more (or less) toxic compounds; (5) distribution in body fluids (protein-binding, lipophilicity, and hydrophilicity); (6) activity of naturally occurring inhibitors or promoters; (7) metabolic rate in active sites (Glassock, 2008).

All uremic compounds play a negative role in biological functions, causing many signs and symptoms seen in people with CKD. Protein-bound compounds are known as difficult-to-remove cytotoxic molecules which promote numerous deleterious effects in various tissues, including the cardiovascular system. Uremic toxins, especially those of low molecular weight, bind to proteins after being absorbed, mainly to Human Serum Albumin (HSA), and are not always effectively removed from the body of CKD patients through peritoneal dialysis or hemodialysis (Ito et al., 2010; Watanabe et al., 2013; Vanholder et al., 2014). We can highlight indoxyl sulfate (IS), phenyl sulfate, indole-3 acetic acid, uric acid, *p*-cresol (PC), *p*-cresyl sulfate (PCS), and homocysteine among the uremic compounds bounded to proteins which have been extensively explored (Kikuchi et al., 2010; Watanabe et al., 2012; Poveda et al., 2014).

## Uremic Toxins of Low Molecular Weight

### Small and Soluble in Water Compounds

According to Duranton et al. (2012) there are 88 uremic solutes and free water-soluble (<500 Da) solutes represent 46% of the total. Water-soluble compounds almost never bind to proteins and many of the soluble toxins are already well known and studied, such as creatinine, urea and cytokines. Other compounds, which will be discussed further on, are also important, but have only recently gained attention (Glorieux et al., 2004).

One class of water-soluble compounds which is important to mention is guanidino compounds, which includes guanidine, guanidinosuccinic acid, creatinine, and others. It has been demonstrated that these compounds have neurotoxic effects (De Deyn et al., 2001) and play a role in potentially damaging the vessels, as they seem to stimulate leukocyte function, as shown in an *in vitro* study by Glorieux et al. (2004).

For example, creatinine and urea were observed to cause cardiomyocyte contractile injury, as well as increase cardiac oxygen consumption by lowering norepinephrine and cause insulin resistance (Lekawanvijit and Krum, 2015). Uric acid is also associated to heart injury as it is increased during hypertension, atrial fibrillation and heart failure (Tamariz et al., 2014). Uric acid was previously described to cause adverse cardiovascular (CV) effects such as oxidative stress, inflammation, endothelial dysfunction and increase on renin-angiotensin-aldosterone system (RAAS) activity after stimulating cardiomyocytes (Chaudhary et al., 2013). Moreover, several studies have linked high levels of uric acid as a risk factor for the progression of CKD in type 2 diabetes, in addition to being associated with an increased risk of hypertension and CVD. The mechanism that uric acid contributes to these comorbidities is not yet fully understood, but pharmacological interventions capable of reducing urate production or increasing urate excretion in hyperuricemic patients have consistently improved the cardiorenal prognosis (Kalra et al., 2020).

Trimethylamine *N*-oxide (TMAO) is a water-soluble low molecular weight uremic toxin from the amine group derived from dietary choline, phosphatidylcholine, L-carnitine, and betaine (Craciun and Balskus, 2012). TMAO has been shown to accumulate in the plasma of CKD patients (Bain et al., 2006), and increased TMAO concentrations are correlated with coronary atherosclerosis (Stubbs et al., 2016). Most studies correlate TMAO with cardiovascular events; however, some studies have shown that TMAO plays a pivotal role in renal fibrosis (Tang et al., 2014). Regarding atherosclerosis, evidence has shown that TMAO promotes inflammation through activation of the NLRP3 (Nucleotide-binding oligomerization domain leucine-rich repeat proteins 3) inflammasome and consequently endothelial dysfunction (Boini et al., 2017).

The inorganic phosphorus (Pi) is also a small-studied molecule, even though it is not considered a uremic toxin for some authors, its importance for the progression of CVDs is extremely notorious. Although increased serum Pi levels are already seen at the onset of kidney disease, renal Pi excretion becomes insufficient in advanced CRF to remove phosphorus that is consumed daily from the diet. Moreover, dialysis practice is not able to repair serum phosphorus levels, causing hyperphosphatemia usually observed in renal failure patients, particularly those on dialysis (Block et al., 1998; Barreto et al., 2014). Phosphorus metabolism disorders have other important unfavorable consequences. Besides to accelerating the progression of renal dysfunction, hyperphosphatemia is related to the pathogenesis of secondary hyperparathyroidism (Sampaio et al., 2008). Moreover, studies have connected hyperphosphatemia to an extensively higher incidence of mortality from CVD, as well as mortality from peripheral and visceral vascular calcification, observed in patients with CKD (Dhingra et al., 2010; Cancela et al., 2012).

The relationship of Pi with CVDs, such as ventricular hypertrophy, coronary disease and heart failure, increases the mortality rate, placing this compound among the uremic toxins with the greatest deleterious effect on the cardiovascular system (Tonelli et al., 2005; Dhingra et al., 2010; Barreto et al., 2014). Pi has been pointed out in several studies as a uremic toxin which causes cardiovascular calcification (Holmar et al., 2020).

Dialysis patients have demonstrated a positive correlation between high levels of serum Pi and vascular calcification with mortality rate. A retrospective cohort study conducted on a population of 3,490 performed serum phosphate measurements during the previous 18 months. This study showed that serum phosphate levels >3.5 mg/dL were associated with higher risk of death, and linearly increased with each subsequent 0.5 mg/dL increased serum Pi level (Kestenbaum et al., 2005). Block et al. (1998) demonstrated that elevated serum phosphate levels were associated with an increased risk of death among the population of CKD patients independent of PTH.

The effect of hyperphosphatemia on vascular calcification is a substantial factor for CVDs progression in patients with uremic hyperphosphatemia. It was observed an increase on expression of vascular cells from osteogenic genes such as osteopontin (OPN) and bone morphogenetic protein (BMP-2) that stimulate osteogenic conversion of smooth muscle cells. Studies in mouse cell cultures showed that a medium with high phosphate content increased the expression of BMP-2 and OPN in smooth muscle cells, being responsible for calcification of the vascular bed (Sage et al., 2011). In addition, Belmokhtar et al. (2019) demonstrated that the receptor for advanced glycation end products (RAGE) and a potential modulator of the sodium phosphate co-transporter through the modulation of phosphate inorganic transporter 1 (Pit-1) expression, is a key protein in triggering vascular calcification during high Pi concentrations. In an experimental model of mice with chronic kidney failure, Phan et al. (2008) found that carbonate supplementation decreased vascular calcification, being consistent with the hypothesis of a predominant role of phosphorus over calcium in promoting vascular calcification in CRF.

The toxic effects of Pi present dangers to the myocardium. Dhingra et al. (2010) performed an echocardiographic study which correlated the serum phosphorus levels to left ventricle (LV) cross-section measurements and to heart failure in 3,300 participants with CKD. Their results showed that serum phosphorus was positively related to the internal mass of the left ventricle and systolic dysfunction. A total of 157 individuals developed heart failure after monitoring the patients for 17 years. Their adjustment models for established risk indicate that increase on serum phosphorus levels was directly associated with risk of heart failure (Dhingra et al., 2010).

Clinical evidence points to Pi as playing a fundamental role in the development of CVDs. Given that CVDs are the principal cause of death worldwide, these findings present an eminent concern in relation to public health, as Pi has been used in the food industry as a food additive, being widely ingested in the Western diet.

### Medium Compounds

Medium uremic toxins have moderately high molecular weight (>500 Da) and are represented by β_2_- microglobulin and pro-inflammatory molecules such as interleukin-6 (IL-6), β-trace protein (BTP), pentatriz-3, and parathyroid hormone (PTH) (Barreto et al., 2014). Others medium compounds are more intensively studied as CKD biomarkers such as fibroblast growth factor 23 (FGF-23), since it is a possible active agent with a therapeutic target (Massy and Liabeuf, 2017).

Although some dialysis membranes allow the passage of medium compounds (Duranton et al., 2012) various medium molecules contribute to the high mortality and morbidity rate observed in CKD patients (Massy and Liabeuf, 2017). Neirynck et al. (2012) points out that the glomerular filtration rate (GFR) is a weak indicator of the levels of these uremic solutes in renal diseases. Thus, its use to assess renal function does not reflect the concentration of several uremic solutes with a proven toxic impact on CKD.

Many of these compounds are peptides and show a strong independent association with adverse clinical results, affecting a large number of organs and systems (Barreto et al., 2014). However, in addition to those of risk which have been widely described in the literature, these uremic compounds can be promising candidates for treatment through their elimination by dialysis techniques or pharmacological therapies (Fliser et al., 2007; Titan et al., 2011; Massy and Liabeuf, 2017).

Elevated levels of medium toxins such as FGF-23 and PTH contributes to progressive renal structural damage, strongly interfering in the progressive reduction of GFR and cardiac function. It is interesting to point that these mentioned toxins are naturally produced by the organism and are fundamental to maintaining mineral homeostasis in the body.

FGF-23 is a medium protein of 32kDa and produced by bone cells, specifically, osteocytes and osteoblasts. It accumulates in the plasma if not filtered during dialysis, causing a phosphate disequilibrium since its levels physiologically rises in parallel to a decrease the serum phosphate excess (Kuczera et al., 2016). Elevated plasmatic FGF-23 can also cause cardiovascular complications since it was able to promote cardiomyocytes hypertrophy *in vivo* and *in vitro* (Faul et al., 2011). When stimulated with FGF-23, cardiomyocytes suffer contractile dysfunctions and intracellular calcium disequilibrium, in addition to being hypertrophied (Navarro-García et al., 2019).

FGF-23 action reaches the parathyroid gland, suppressing PTH production in the parathyroid gland. In contrast, the excess PTH reduces FGF-23 production, leading to a negative feedback loop (Navarro-García et al., 2018). PTH is a 94 kDa molecular weight peptide and it is responsible for inhibition of phosphate reabsorption in renal proximal tubular cells and also upregulates the 1α-hydroxylase gene, capable for conversion of 25-hydroxyvitamin D to the active metabolite of vitamin D [1,25-dihydroxyvitamin D (1,25[OH]_2_D_3_)]. PTH is also responsible for increasing the calcium reabsorption by insetting calcium channels in the apical membrane of distal tubules (Duque et al., 2020). PTH-induced cardiomyocyte hypertrophy may be an indirect effect of FGF-23 once high levels of FGF-23 have also been bonded to cardiac hypertrophy and mortality in CKD patients (Duque et al., 2020).

Uremic patients frequently present mineral bone disorder (MBD) which is a systemic syndrome characterized by vascular calcification, abnormalities in bone turnover, vitamin D deficiency, defective metabolism of calcium and phosphate, an increase in FGF-23 and PTH levels (Duque et al., 2020). In CKD-MBD patients, plasma FGF-23 concentration increases due to significant changes in phosphate or serum PTH concentration. Kuczera et al. (2016) consider FGF-23 and PTH as secondary uremic toxins, as they only increase after phosphate accumulation, even if it is minimum. Also, the decreased renal clearance of FGF-23 due to a decrease on glomerular filtration can contribute to an increase in FGF-23 plasmatic (Kuczera et al., 2016).

Hyperphosphatemia has as consequences the secondary hyperparathyroidism and the propensity to metastatic calcification when the serum calcium and phosphorus (Ca × PO_4_) is elevated (Block et al., 1998). Thereby, the control of phosphorus concentrations has long been noted as an essential factor in the clinical conduct of patients with renal failure (Delmez and Slatopolsky, 1992).

The study carried out by Neves et al. (2004) demonstrated an association of renal failure, myocardial hypertrophy and prejudicial effects on bone remodeling followed by hyperphosphatemia. These conditions were not corrected by PTH treatment, emphasizing the relevance of phosphorus in reducing mortality and morbidity in chronic renal patients (Neves et al., 2004). In addition, when using sevelamer, a ligand that prevents the absorption of Pi, Chertow et al. (2002) found a lower probability of causing hypercalcemia, low PTH levels and progressive coronary aortic calcification in CKD patients. These findings were corroborated by Graciolli et al. (2009) who evidenced that phosphate is a critical factor for vascular calcification, revealing that hyperphosphatemia, even without an initial association with CVD, is correlated with upregulation of runt-related transcription factor 2 (Runx2) gene in vascular smooth muscle cells (VSMCs). Treatment with sevelamer was also associated with cardiovascular improvement showed by reducing aortic stiffness, diastolic dysfunction and cardiac hypertrophy in chronic renal failure (CRF) model mice (Maizel et al., 2013).

As mentioned, Pi levels increase when renal function decreases, and thereafter promote secretion of the FGF-23 in urine. In the beginning of CKD, FGF-23 attenuates vascular calcification, preventing an increase in serum phosphate levels, while FGF-23 is unable to sustain normal phosphate levels in end-stage renal disease. Therefore, high FGF-23 levels and hyperphosphatemia are associated to hypertension, cardiac hypertrophy and vascular calcification (Faul et al., 2011; Navarro-García et al., 2018). Important findings regarding serum Pi levels and cardiovascular morphological responses showed a direct effect of Pi on the heart, inducing hypertrophy of the myocardium, hyperplasia of cardiomyocytes and interstitial fibrosis and vessels, even without changes in PTH levels (Barreto et al., 2014).

## Protein-Bound Uremic Toxins (PBUT)

Protein-bound compounds are cytotoxic molecules which have shown deleterious effects in various tissues, including the cardiovascular system (Barreto et al., 2014). Phenols and indoles are highlighted among these compounds as being of low molecular weight which join proteins present in blood plasma for transport, especially serum albumin (Barreto et al., 2014). The principal protein-bound uremic toxins (PBUT) are Indoxyl Sulfate (IS), a derivative of tryptophan; and *P*-Cresyl (PCS), a toxin derived from tyrosine. These toxins have low molecular weight (213 and 108 Da, respectively) and a strong affinity for serum proteins, which makes their removal by dialysis difficult (Niwa, 2010). Next, we will approach the IS and PCS compounds, highlighting the scientific findings about these uremic retention compounds and their deleterious effects on CKD.

**FIGURE 1:**
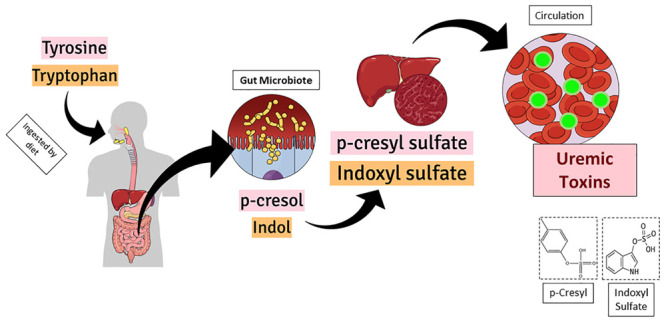
Graphical representation of *p*-cresyl (PCS) and indoxyl sulfate (IS) formation inside the body. Both uremic toxins, *p*-cresyl sulfate and indoxyl sulfate are originate from the intestinal microbial metabolism of dietary amino acids. While the IS is delivered from the tryptophan, the PCS is delivered from tyrosine. After the chemical modifications occurred in the liver, both active metabolites reach the circulation and impact target organs.

Figure 1 shows the graphical representation of *p*-cresyl (PCS) and indoxyl sulfate (IS) formation inside the body. Both uremic toxins, *p*-cresyl sulfate and indoxyl sulfate are originate from the intestinal microbial metabolism of dietary amino acids. The IS is generated from the tryptophan and the PCS is delivered from tyrosine. After the chemical modifications occurred in the liver, both active metabolites reach the circulation and impact target organs (Lesaffer et al., 2000; Schepers et al., 2006). Thus, prophylactic actions such as preserving renal function and decreasing production of uremic toxins, are needed to maintain these protein-bound compounds low (Barreto et al., 2014; Gryp et al., 2017).

### Indoxyl Sulfate (IS)

Indoxyl Sulfate (IS) is a uremic compound resulting from a low molecular weight protein diet. It is a derivative of the amino acid tryptophan which is degraded by intestinal bacteria. In blood plasma it is primarily bound to the protein albumin, and this binding causes its excretion to primarily occur by proximal tubular secretion and then by glomerular filtration (Lekawanvijit, 2018). Organic anionic transporters (OATs) interact with several endogenous substances in tubular cells, such as IS. Thus, IS is captured by the blood in the basolateral membrane by organic anion transporters (OATs), OAT1 and OAT3, and accumulates in the cells when in high concentrations (Taki et al., 2006). CKD patients often present a total IS concentration which exceeds 500 μM compared to 0.1–2.39 μM in healthy patients (Bolati et al., 2013).

Conventional hemodialysis is not effective for removing IS, as 90% of the IS is bound to albumin and the complete molecular IS-albumin is larger than the dialysis membrane pore. In addition, the IS concentration in free or protein-bound form is approximately 1–9, which provides a circulating IS reservoir even after dialysis in uremic patients. The IS retained in CKD is also associated with several harmful effects on other organs, such as changes in thyroid function, endothelial dysfunction, hyperplasia of smooth muscle cells, vascular calcification and increase in atherosclerosis in men (Gao and Liu, 2017; Holmar et al., 2020).

*In vitro* studies have shown that IS has a hypertrophic effect in cardiomyocyte cultures by activating the signaling of mitogen-activated protein kinase (MAPK) and NFκB pathways, indicating that IS can play a critical role in the development of hypertrophy conditioned uremic conditions (Kim et al., 2019). In addition, an accumulation of IS can cause degradation of the remaining renal nephrons, mainly in the proximal tubular cells, stimulating glomerular sclerosis, renal fibrosis and the progression of CKD contributing to increase the expression of the pro-α1 collagen, transforming growth factor β1 (TGF-β1) and tissue inhibitor of metalloproteinase 1 (TIMP-1) genes, resulting in greater loss of nephrons, which in turn increases the CKD progress (Niwa, 2010). Moreover, other studies have indicated a rise in the levels of pro-inflammatory cytokines such as IL-6, its link with coronary artery diseases and vascular injury, thus contributing to accentuate CKD progression and the mortality rate in this population (Mozar et al., 2011; Wu et al., 2011).

In this context, Nakano et al. (2019) suggests that there is an immune dysfunction caused by IS; the activation of pro-inflammatory macrophages is mediated by their uptake through transporters, including OATP2B1, which is an important mediator of the inflammatory process. In addition, Kim et al. (2019) demonstrated the interaction between the aryl hydrocarbon receptor (AhR), nuclear factor kappa-light-chain-enhancer of activated B cells (NF-κB) and the suppressor cytokine signaling (SOCS) 2, which is important for the production of TNF-α in human macrophages stimulated by IS.

Scientific findings have shown that increases in IS concentrations which occur from the beginning of CKD progression are capable of activating the deposition of fibroblasts via the HSP90 depletion pathway or TGF-β1 induction mediators, and similar gene matrices against decapentaplegic (SMAD3), contributing to renal fibrosis, thus increasing the pro-inflammatory phenotype and progressing to renal degradation (Shimizu et al., 2013). In addition, increasing IS concentrations can induce renal and vascular cell senescence, and increase reactive oxygen species (ROS), contributing to increased oxidative stress (Bolati et al., 2013; Itoh et al., 2013). Increased IS concentrations in the endothelium can cause a release of microparticles, inhibition of cell proliferation and repair, in addition to increasing leukocyte interaction/adhesion causing endothelial enlargement (Dou et al., 2004; Faure et al., 2006). The effects of IS for cardiac tissue generally cause increased inflammation, cardiac fibrosis, proliferation of cardiomyocytes, in addition to increasing cardiomyocyte hypertrophy and oxidative stress in the heart (Yisireyili et al., 2013).

### *P*-Cresyl Sulfate (PCS)

The *P*-cresol (4-methylphenol) uremic toxin has low molecular weight (108 Da) generated from the metabolism of tyrosine and phenylalanine, two aromatic amino acids which are metabolized by putrefactive bacteria from the intestinal microbiota (Koppe et al., 2013; Barreto et al., 2014). *P*-cresol is processed through sulfation and glucuronidation by the action of the sulfotransferase enzyme contained in the cell cytoplasm as it passes through the mucosa in the distal part of the colon of the large intestine and liver; this process then generates two compounds, PCS and *p*-cresyl-glucoronate. Although these substances are found in their conjugated and non-conjugated forms in CKD patients, their biochemical impacts are different (Gryp et al., 2017).

*P*-cresol is present in small concentrations in the human body, as it is promptly metabolized and is not detected in uremic patients, or normal people in its unconjugated form. Thus, PCS (C_7_H_8_O_4_S) is the conjugated form of the *P*-cresol with evident retention in uremic patients, which circulates in the bloodstream in CKD patients (Koppe et al., 2013; Barreto et al., 2014; Vanholder et al., 2014).

About 95% of the PCS solute reversibly binds to plasma albumin in circulating blood, and soon a balance is established between the bound fractions and the free fractions of this compound in the blood. The free fractions are filtered directly into the renal glomeruli, while their protein-bound fraction is eliminated by the tubular epithelial cells in the kidney’s proximal tubules. However, both fractions (linked to albumin or free) are excreted in the form of urine (Gryp et al., 2017).

In addition, it is known that dialysis and non-dialysis CKD patients usually present reduced protein assimilation in the small intestine, and this condition tends to increase with the worsening of the renal situation. In this sense, protein malnutrition can further increase the PCS levels metabolized in the colon by proteolytic bacteria. A decrease in protein assimilation may be related to metabolic diseases such as diabetes mellitus, gastrointestinal disorders (among other diseases), or protein malnutrition related to diet [a common situation for dialysis patients, with CKD progression tending to increase in all of these cases (Vanholder et al., 2001; Neirynck et al., 2013)].

*p*-cresyl sulfate mean serum concentrations in individuals without renal impairment vary between 2.8 ± 1.7 and 6.6 ± 3.7 mg/L measured by ultra-performance liquid chromatography (UPLC) and mass spectrometry (MS), respectively. In addition, serum concentrations in individuals with end-stage CKD can vary from 21.8 ± 12.4 to 106.9 ± 44.6 mg/L evaluated by UPLC and LC-MS, in plasma, respectively (Vanholder et al., 2014).

The biological effects of PCS have been reported in the literature since 2005. Several *in vivo* studies with human and animal models and *in vitro* have shown a considerable range of harmful biological activities to the organism and mainly to the cardiovascular system (Gryp et al., 2017). There is evidence that the increase in PCS in the kidneys causes increases in the expression of cytokines and pro-inflammatory genes in renal tubular cells, in addition to RAAS activation and epithelial-mesenchymal transition, fibrosis, and nephrosclerosis (Sun et al., 2012b, 2013). In addition to renal tubular damage, the increase in PCS levels is related to the decrease in Klotho through the methylation of the Klotho gene, contributing to the senescence of renal cells (Sun et al., 2012b; Watanabe et al., 2013).

Studies involving the accumulation of PCS in CKD have shown a wide range of harmful effects of this toxin in the body, with the production of free radicals and leukocyte activation among them, interfering in the inflammatory response (Schepers et al., 2006; Gondouin et al., 2013; Watanabe et al., 2013). In addition to vascular damage induced by inflammation, PCS is able to induce the release of microparticles from endothelial cells even in the absence of primary injury (Meijers et al., 2009).

There is evidence that the toxicity resulting from PCS accumulation is associated with installing altered metabolic conditions such as insulin resistance and ectopic lipid dysfunctions in the muscles and liver (Koppe et al., 2013). In addition, effects resulting from this toxicity can be observed in the kidneys, such as induced toxic and pro-apoptotic effects of proximal tubular cells and renal fibrosis (Bammens et al., 2003; Mozar et al., 2011; Neirynck et al., 2013). It has also been demonstrated that PCS activates the renal RAAS, TGF-β1pathway and induces epithelial-to-mesenchymal transition-like transition leading to renal fibrosis (Sun et al., 2012b). PCS uremic concentrations in the vascular endothelium have been linked to the release of microparticles determining endothelial damage, inducing oxidative stress in human vascular smooth muscle cells (HVSMCs) and human umbilical veins endothelial cells (HUVECs) (Meijers et al., 2009; Pletinck et al., 2013; Gross et al., 2015).

In *in vivo* experiments, PCS is capable to induced contraction of thoracic aorta through rho-kinase signaling, independent of oxidative stress and internal vascular remodeling (Gross et al., 2015). Besides, PCS increased induce an increase on nicotinamide adenine dinucleotide phosphate (NADPH) oxidase activity and production of ROS in cardiac myocytes, contributing to apoptosis and diastolic dysfunction in nephrectomy model in mice (Han et al., 2015). Given this, we can highlight the association of the increase in this compound in CKD patients with a high mortality rate (Poveda et al., 2014).

## Intracellular Mechanisms Involved in Protein-Bound Uremic Toxin Signaling: Aryl Hydrocarbon Receptor (AhR)

It is already known that uremic toxins are cardiotoxic. In fact, most of the CVDs promoted by these toxins are related to cardiovascular complications associated to CKD through their accumulation and activation of pro-oxidative/inflammatory pathways due to human AhR. However, increased cardiovascular risks under non-uremic conditions are also associated to AhR activation (Sallée et al., 2014).

The cellular message carried by uremic toxins, mainly IS or indole-3-acetic acid (IAA), has significant importance as it triggers responses which can change the activity of many genes or even induce an entire process such as cell division or apoptosis. PBUTs have been recognized as endogenous agonists for the AhR (Schroeder et al., 2010). This cytoplasmatic receptor is widely expressed in the human body and regulates numerous genes, encoding xenobiotic enzymes such as the P450 1A1 cytochrome (*Cyp1a1*) (Hankinson, 1995).

There are two described AhR signaling pathways: the canonical and non-canonical. The canonical pathway is based on Cyp1a1 gene activation. Without stimulus, the inactive state of AhR is normally in cytoplasm conjugated with HSP90 and the co-chaperone p23. Once it is activated by a ligand, it changes its conformal structure and migrates to the nucleus and binds to AhR (Lamas et al., 2018). The complex formed activates the genomic region called dioxin response element (DRE) where the Cyp1a1 and AhR repressor genes are coded such as *Cyp1a2* and *Cyp1b1*, glutathione-*S*-transferase A, NAD(P)H, quinone oxidoreductase 1, uridine 5′–diphosphate-glucuronosyltransferase 1A and aldehyde dehydrogenase-3 (Lamas et al., 2018).

The non-canonical AhR signaling interact directly with the retinoblastoma protein (pRb) which are responsible for blocking the cell cycle and suppressing the S-phase genes. After the ligand (uremic toxin) binding, AhR can react with the hypophosphorylated pRb, arresting the cell cycle in the G1 stage. There are studies which point to potential carcinogenesis control due to this interaction. Thus, there an induction of inflammatory cytokine expression is given through the interaction of AhR with NF-kB, which in turn promotes a decrease in the expression of Cyp1a1. This ligand activation can also promote proteolysis of the endoplasmic reticulum (ER), assembling the ubiquitin ligase complex. After promoting this bonding, the AhR binds to genomic regions containing DRE, regulating the target genes (Lamas et al., 2018).

AhR is a promiscuous receptor since it is probable to bind to a rage of endogenous and exogenous ligands. Nevertheless, its canonical signaling pathway is narrowly controlled by three different checkpoints: (1) clearance of ligands by CYP1A1; (2) proteasomal degradation; and (3) cleavage of the AhR and AhR nuclear translocator (AhR/ARNT) complex (Stockinger et al., 2014).

The expression of AhR is detected in different tissues such as the heart, intestine, brain, lungs and kidney, having been identified in greater quantity in the pulmonary microvasculature and aortic arch. The cardiac endogenous function of AhR has already been characterized using knockout mice (AhR gene deficient mice), including in heart function, vascular development and blood pressure (Dolwick et al., 1993).

Regarding CKD patients, the most relevant AhR ligands are uremic toxins which are generated by tryptophan metabolism. As mentioned earlier, tryptophan metabolism involves the indolic pathway in which the gut microbiota transforms tryptophan into indoxyl and after indoxyl sulfate; or in indole-3-acetaldehyde, indole-3-acetaldehyde, indole-3-propionic acid and IAA (Zelante et al., 2013), generating metabolites which represent powerful AhR ligands. CKD patients are subjected to a high amount of uremic toxins daily, causing a higher risk of CVD. It has been showed that AhR is more stimulated in CKD stage 3 patients and is intimately linked to the IS level and are inversely proportional to the epidermal growth factor receptor (eGRF) (Dou et al., 2018). In addition, the genetic targets such as Cyp1a1 and AhR are also upregulated in CKD patients as well as in 5/6 nephrectomized mice (Dou et al., 2018). This same study reported that CKD is extremely attenuated in AhR deficient mice threated with IS (Dou et al., 2018).

Glomerular injury caused by IS is constituted by the AhR activation in podocytes. This activation triggers proinflammatory phenotype injuring both *in vivo* and *in vitro* podocytes (Ichii et al., 2014). The IS accumulation due to CKD switches off the endothelial proliferation, triggering the oxidative stress in renal tissue (Brito et al., 2017). In a mice model of CKD, IS was able to affects the iron metabolism by the AhR regulation of the oxidative stress pathways. Not only IS can lead to CKD progression, as IAA can also promote an AhR response. This uremic toxin activates the intracellular AhR/p38MAPK/NF-κB signaling, leading to an increase on cyclooxygenase-2 (COX-2) expression and production of ROS in experimental models *in vivo* and *in vitro* (Dou et al., 2015). Leukocyte activation during CKD is also triggered by thrombosis and inflammation enhanced by vascular oxidative stress (Roman et al., 2018). AhR contributes to the progression of AKI into CKD given the previously cited pathway activation.

AhR inhibition is fundamental in the context of cardiorenal protection during the accumulation of uremic toxins. The IS-AhR-mediated cardiac injury stimulates the activator protein-1 gene expression, increasing E-selectin gene expression, inflammation and leukocyte adhesion into the vascular endothelial cells. This mechanism also intensifies the vascular calcification in the early stages of arteriosclerosis (Sallée et al., 2014). The increase on myocardial infarction risk and peripheral artery diseases in CKD patients are mediated by vascular dysfunction caused by AhR activation that may lead to atherosclerotic thrombosis (Zhao et al., 2019).

Furthermore, the AhR activation can also react with the Wnt/β-catenin, transforming growth factor-β/BMP-2 and NOTCH signaling pathways, beyond tyrosine kinase pathways, including receptor of growth factors such as keratinocyte, epidermal and vascular endothelial, in some human diseases (Roman et al., 2018). Wnt/β-catenin signaling pathway exerts an important function on embryonic cardiac development as well as adult heart homoeostasis. Thus, changes in this signaling pathway are linked to a variety of cardiac disease conditions, including fibrosis, arrhythmias, hypertrophy and infarction (Park et al., 2018).

## Uremic Toxins in Endothelial Dysfunction

Uremic toxins are closely related to endothelial dysfunction and the development of CVDs in CKD patients (Zhu et al., 2016; Wu et al., 2021). Studies have shown that uremic toxins deregulate endothelial functions, causing structural damage, inflammation and impaired endothelium-dependent vasodilatation (Zhu et al., 2016; Vila Cuenca et al., 2019). Indeed, poor microvascular endothelial function is associated with CKD progression as well as albuminuria (Seliger et al., 2016). CKD patients on hemodialysis and on peritoneal dialysis importantly have a similar endothelial response, which is significantly reduced compared to a healthy group (Alexandrou et al., 2020). It is worth noting that in addition to uremic toxins, other factors such as shear stress may also contribute to endothelial dysfunction in CKD patients (Boulanger et al., 2007; Verbeke et al., 2007).

Transporters or receptors may act like a mediator between uremic toxins and endothelial cells. Cellular uptake of PCS and IS has been linked to OATs such as OAT1 and OAT3 (Miyamoto et al., 2011; Gregorio et al., 2018; Li et al., 2018). It was observed *in vitro* that the PCS and IS uptake by endothelial cells was decreased in the presence of probenecid, an OAT inhibitor (Favretto et al., 2017). On the other hand, Pi cell transport is mediated by the sodium-dependent phosphate cotransporters (NaPiTs), especially PiT-1 and Pit-2 (Abbasian et al., 2015; Inden et al., 2016). In fact, inhibition or silencing of PiT-1 expression reverses the increase of inorganic phosphate intracellular levels in endothelial cells subjected to hyperphosphatemia (Abbasian et al., 2015). In contrast, advanced glycation end products (AGEs) interact with the receptor for AGEs (RAGE) in the cell membrane, triggering several pathways which lead to endothelial dysfunction (Stinghen et al., 2016; Gregorio et al., 2018; Wang et al., 2019).

Uremic toxins compromise cell-to-cell communication by disrupting cell junctions and causing loss of integrity in the endothelial barrier. Inorganic phosphate and uremic serum decrease the expression of vascular endothelial (VE)-cadherin and zonula occludens-1 (ZO-1), constituent proteins of cell-to-cell junctions (Maciel et al., 2018). PCS and IS induce VE-cadherin phosphorylation, which may lead to its disassembly and cell internalization (Shang et al., 2017; Assefa et al., 2019). In addition to the loss of cell-to-cell junctions, uremic serum also induces cytoskeleton remodeling and changes in cell morphology (Peng et al., 2012a; Maciel et al., 2018; Vila Cuenca et al., 2019). The main consequences of structural endothelial damage are the increased vascular permeability and vessel leakage (Chistiakov et al., 2015; Claesson-Welsh et al., 2020), several studies have shown that PCS, IS, AGEs and uremic serum increase endothelial barrier permeability (Hirose et al., 2010; Yang J. et al., 2015; Maciel et al., 2018; Assefa et al., 2019; Vila Cuenca et al., 2019; Chen et al., 2020).

The glycocalyx of the endothelium is also damaged in CKD, which is evidenced by the excessive shedding of its components such as syndecan-1, hyaluronan and heparan sulfate. Clinical studies have demonstrated that syndecan-1 and hyaluronan levels are elevated in the serum of patients with advanced CKD and are correlated with PCS and IS serum levels (Vlahu et al., 2012; Padberg et al., 2014; Liew et al., 2021). An increase in syndecan-1 serum levels and a reduction in the thickness of the endothelial glycocalyx have been shown in an animal model of CKD (Padberg et al., 2014). Likewise, IS-treated rats showed an increase in heparan sulfate serum levels, while there was a decrease in its detection in the tissue (Gondouin et al., 2013). These data suggest that there is a degradation of endothelial glycocalyx in CKD, which may promote leukocyte adhesion and vascular inflammation (Gondouin et al., 2013; Delgadillo et al., 2020).

**FIGURE 2:**
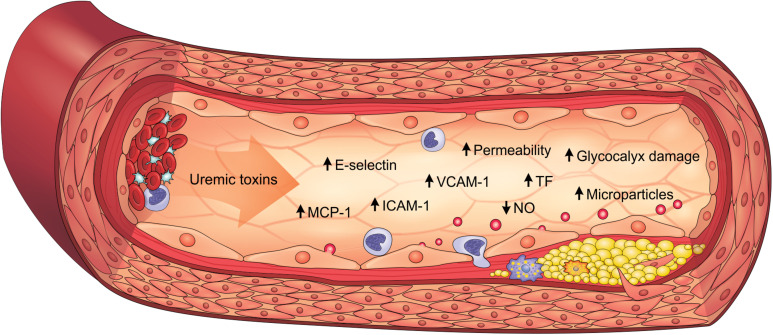
Uremic toxicity is linked to endothelial dysfunction in CKD. Uremic toxins induce the expression of proinflammatory factors (e.g., MCP-1, E-selectin, ICAM-1, and VCAM-1), prothrombotic factors (e.g., TF), damage to the glycocalyx, the increase in permeability, the reduction of NO bioavailability and the formation of endothelial microparticles. As a result, endothelial dysfunction contributes to the pathogenesis of cardiovascular diseases, such as atherosclerosis. ICAM-1, intercellular adhesion molecule-1; MCP-1, monocyte chemoattractant protein-1; NO, nitric oxide; TF, tissue factor; VCAM-1, vascular cell adhesion molecule-1.

Inflammatory activity is enhanced in the arterial walls of CKD patients and strongly associated to cardiovascular outcomes such as atherosclerosis (Bernelot Moens et al., 2017). Indeed, endothelial cells play an important role in the expression of proinflammatory cytokines, chemokines, and adhesion molecules, which is heightened by uremic toxicity. PCS, IS, AGEs and uremic serum increased the expression of monocyte chemoattractant protein-1 (MCP-1) *in vitro*, constituting an important molecule for monocyte recruitment (Stinghen et al., 2009; Yang et al., 2012; Jing et al., 2016; Favretto et al., 2017; Maciel et al., 2018). These uremic toxins similarly also induced E-selectin, vascular cell adhesion molecule 1 (VCAM-1) and intercellular adhesion molecule 1 (ICAM-1) expression, which mediate leukocyte adhesion and transendothelial migration (Stinghen et al., 2009; Rempel et al., 2015; Ito et al., 2016). Corroborating these findings, E-selectin, VCAM-1, ICAM-1, MCP-1 and TNF-α levels are elevated in the aortic tissue of *ApoE^–/–^* mice with CKD, as well as in PCS-treated animals (Watanabe et al., 2013; Han et al., 2016; Jing et al., 2016). Clinical studies have also shown that these proinflammatory factors are elevated in CKD patients, particularly in the more advanced stages (Stinghen et al., 2009; Gupta et al., 2012). As a result, leukocytes are activated and recruited to the endothelium, which contributes to the atherogenesis process. Accordingly, studies have shown that PCS and IS increase interactions between leukocytes and endothelium (Ito et al., 2010; Han et al., 2016; Jing et al., 2016). Notably, atherosclerotic lesions and greater macrophage infiltrate are observed in the aortas of mice with CKD, an effect which is enhanced with PCS (Jing et al., 2016). Therefore, uremic toxins play a prominent role in inducing the proinflammatory state of the endothelium in CKD (Figure 2).

Uremic toxins cause impaired endothelium-dependent vasodilatation, particularly due to the decrease in bioavailability of nitric oxide (NO), a potent vasodilator and a hallmark of endothelial dysfunction. IS and inorganic phosphate reduced NO levels in endothelial cells *in vitro* (Tumur and Niwa, 2009; Stevens et al., 2017). NO production in these cells is mediated by endothelial nitric oxide synthase (eNOS), whose activity and expression are decreased by uremic serum, IS, AGEs, hippurate and trimethylamine-*N*-oxide (TMAO) (Ren et al., 2017; Shang et al., 2017; Huang et al., 2018; Wu et al., 2021). Asymmetric dimethylarginine (ADMA), another uremic toxin, also inhibits eNOS (Kajimoto et al., 2012) ADMA is an endogenous analog of L-arginine. L-arginine is the substrate for eNOS and ADMA inhibits this enzyme activity, causing endothelial damage. Furthermore, endothelium-mediated relaxation as well as vascular eNOS activity were impaired in an animal model of CKD (Kajimoto et al., 2012; Huang et al., 2018). The pro and antithrombotic properties of the endothelium are deregulated in CKD. IS and IAA induced procoagulant activity in endothelial cells by increasing tissue factor (TF) expression and consequently the factor Xa formation (Gondouin et al., 2013; Addi et al., 2019). CKD patients similarly have high levels of thrombogenic proteins such as TF, von Willebrand factor (vWF), and thrombomodulin compared to healthy controls (Gondouin et al., 2013; Shivanna et al., 2016; Kaminski et al., 2017). In addition, fibrinolysis markers such as tissue plasminogen activator (t-PA) and plasminogen activator inhibitor-1 (PAI-1) are altered in CKD patients (Kaminski et al., 2017, 2018). These data indicate the compromised coagulation and fibrinolysis mechanisms in CKD. Moreover, elevated procoagulant activity is also induced by microparticles derived from endothelial cells exposed to IS, IAA and inorganic phosphate (Gondouin et al., 2013; Abbasian et al., 2015).

Endothelial microparticles are important for intercellular communication as they carry bioactive molecules from the source cell to recipient cells, including leucocytes and vascular cells (Buendía et al., 2015). Clinical studies have shown that CKD patients have higher levels of endothelial microparticles compared to a healthy group (Faure et al., 2006; Soriano et al., 2014). Furthermore, endothelial microparticles obtained from CKD patients were able to induce VSMC calcification and osteogenesis (Buendía et al., 2015). Endothelial microparticle formation is increased *in vitro* by uremic toxins such as PCS, IS and inorganic phosphate (Faure et al., 2006; Meijers et al., 2009; Abbasian et al., 2015). Endothelial microparticles from cells exposed to IS have elevated PECAM-1 and ICAM-1 levels and were able to modulate endothelial progenitor cells functions (Carmona et al., 2017). In addition, microRNA content in endothelial microparticles are also altered in CKD (Carmona et al., 2017; Shang et al., 2017).

The role of microRNAs in endothelial dysfunction during CKD has been investigated in recent years (Massy and Liabeuf, 2017; Shang et al., 2017). IS, hippuric acid, IAA and uremic serum induced miR-92a expression in endothelial cells, which is associated with reduced expression of endothelial-protective molecules such as eNOS, Krüppel-like factor 2 (KLF2) and KLF4 (Shang et al., 2017). miR-92a levels are also elevated in microparticles isolated from CKD patients compared to a healthy group (Shang et al., 2017). In contrast, CKD patients have decreased miR-126 levels, which is expressed in endothelial cells and has proangiogenic properties (Brigant et al., 2016; Fourdinier et al., 2019). Interestingly, low miR-130a-3p levels were found in microparticles isolated from endothelial cells exposed to IS, as well as from CKD and coronary artery disease patients (Zietzer et al., 2020). IS induced endothelial microparticles *in vitro* with increased levels of miR-181a-5p, miR-4454, and miR-150-5p, which may be related to modulation of the inflammation process (Carmona et al., 2017).

In summary, uremic toxins directly contribute to the pathogenesis of endothelial dysfunction in CKD, being characterized by cells with proinflammatory and prothrombotic properties, endothelium-dependent vasodilatation impairment, microparticle formation, glycocalyx degradation and loss of integrity of the endothelial barrier (Figure 2).

## Implication of Uremic Toxins in the Cardiorenal Syndrome (CRS)

The CRS is a condition in which a heart injury causes a renal injury through the cardiovascular system and vice versa. It is classically divided into 5 types. Type 1 CRS is initially marked by an acute loss of cardiac function, followed by acute kidney injury (AKI) mainly due to hemodynamic changes. The lower renal arterial flow and decrease in the GFR are consequences of acute heart failure observed in type 1 CRS that can be restored when hemodynamic parameters are normalized (Doi and Noiri, 2016). Type 2 CRS occurs due to chronic abnormalities in cardiac function which leads to kidney injury, including disfunctions such as congenital and constrictive cardiac diseases, atrial fibrillation and chronic cardiac ischemia (Doi and Noiri, 2016). Types 3 and 4 are also defined as renocardiac syndrome, acute or chronic, respectively. In type 3, it is possible to observe an AKI, leading to acute cardiac injury. AKI can induce acute cardiac changes by increasing inflammatory processes, oxidative stress and neurohormone secretion (Di Lullo et al., 2017). Type 4 CRS is characterized by cardiovascular commitment in CKD patients representing 70–80% of dialysis patients (Di Lullo et al., 2017). Finally, type 5 is defined by acute or chronic systemic diseases which simultaneously promote cardiac and renal dysfunction at the same time. The factors like sepsis, diabetes, hepato-renal syndrome and diseases related to the immune system have been associated with causes of CRS type 5 (Di Lullo et al., 2017).

**FIGURE 3:**
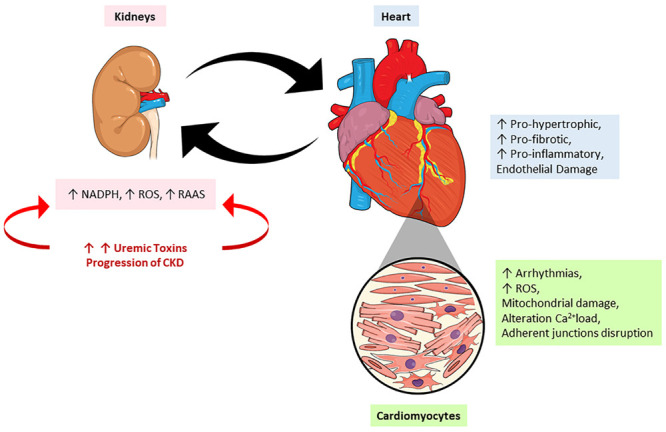
The crosstalk between heart and kidney: contribution of uremic toxins. During the progression of renal lesion there is increase in the circulatory levels of uremic toxins accompanied by an increase on renin-angiotensin-aldosterone system (RAAS) activity and reactive oxygen species (ROS). On the other hand, the heart responds to kidney injury signaling a pro-oxidant, pro-inflammatory effects leading to mitochondrial damage, alterations in calcium loading, arrhythmias and cardiac remodeling. NADPH, nicotinamide adenine dinucleotide phosphate hydrogen; ROS, reactive oxygen species; RAAS, renin-angiotensin-aldosterone system.

The injuries caused by PBUTs are very complex and implicate many pathways which will be discussed soon. According to Di Lullo’s classification, the injury caused by these PBUTs would be classified as type 4 CRS, since it is caused by chronic kidney injury (Di Lullo et al., 2017). It causes permanent damage to the renal tissue, definitively compromising the organ function, which leads to an accumulation of uremic toxins into circulation, thereby causing more damage to the kidney itself, but also to other organs. This can also acutely alter the heart morphophysiology, characterizing type 1 CRS. This injury will mainly cause a decrease in the GFR due to the low arterial flow. Figure 3 mimics the heart-kidney-toxin crosstalk.

The most known outcome of their accumulation is inflammation. IS in cardiomyocytes increases the production of inflammatory cytokines such as interleukin 1 beta (IL1-β), IL-6, and TNF-α (Lekawanvijit and Krum, 2015). This is shown by the previously cited NF-kB interaction. This nuclear factor is the mediator of many effects observed after an accumulation of toxins. The activation of p38 and p44/42 MAPKs by IS seems to present a pro-hypertrophic and pro-fibrotic effect (Fujii et al., 2018). This implies that the cardiac remodeling is activated by IS and suggests the participation of both OAT 1 and 3 (Fujii et al., 2018).

According to Milanesi et al. (2019) mice treated with IS in drinking water showed evidence of tubular necrosis and interstitial fibrosis. This result is supported by the high α-SMA levels expressed in the tissue and the upregulation of HSP90 compared to control mice. The same effects were seen in fibroblast cell cultures due to the upregulation of TGF-β signal and collagen synthesis. The activation of IS-induced fibroblast happens through the HSP 90/Smad 2/3-dependent pathway, as the authors proved by blocking the HSP90 and finding that it causes inhibition of the TGF- β signaling and collagen synthesis (Milanesi et al., 2019). Thus, the renal damage caused by IS is an important target for *in vivo* studies, as well as in different dosages, so the results can be compared and better understood.

In addition, uremic toxins can modulate several signaling pathways causing heart tissue injury, and its action consists of inducing gene expression involved in the inflammatory process and fibrosis in kidney proximal tube cells and oxidative properties in the same cells due to increased NADPH oxidase activity. Moreover, its actuation further leads to macrophage and leukocyte activation and its release of pro-inflammatory cytokines and NF-kB expression in heart tissue (Storino et al., 2015).

As previously mentioned, an accumulation of medium molecules such as FGF-23 and PTH can not only cause serious injury to the heart, but also to other organs. These molecules are fundamental for maintaining a mineral axis and their accumulation leads to effects involving myocardial hypertrophy and contractile disfunction, in addition to muscle weakness, glucose intolerance and osmotic fragility of erythrocytes (Navarro-García et al., 2019; Edmonston and Wolf, 2020).

An accumulation of PBUTs generally increases the risk of death; however, it shows more predisposition in CVDs (Lekawanvijit, 2018). IS and PCS are among the most studied PBUTs. Both are currently being investigated regarding their adverse CV and renal effects. High IS and PCS levels in serum are used to predict CV events and are also implicated with vascular disease, including arteriosclerosis, endothelial inflammation and oxidative stress. Moreover, elevated levels of IS are associated with vascular remodeling, while both PCS and IS are correlated to vascular calcification in different CKD phases (Lekawanvijit, 2018). IS is a highly pro-inflammatory and pro-fibrotic molecule, suggesting that it is directly involved in cardiac remodeling through activation of MAPKs/NF-κB pathway (Lekawanvijit, 2018).

Electrical dysfunction can also occur as a consequence of PBUT’ accumulation. Cardiomyocytes presented an arrhythmogenic profile and also atrial fibrillation *in vivo* when stimulated with IS *in vitro* (Lekawanvijit, 2018). Furthermore, a reduced spontaneous contraction accompanied by an irregular beating of cardiomyocytes and structural and functional changes in communicating junctions were observed after treatment with PCS (Peng et al., 2012b).

## Effect of Uremic Toxins on the Immune System

The alterations on immune system function can have direct or indirect renal effects. In end-stage renal disease (ESRD) is possible to observe that immune function is grievously compromised (Tecklenborg et al., 2018). In addition, CKD patients present an immune system dysfunction caused by dialysis process, vitamin D deficiency and the systemic and sustained inflammatory state induced by higher levels of uremic toxins that leads to an immunosuppression of the innate immune response (Azevedo et al., 2016). The retention of uremic molecules and cytokines activate innate immune response leading to a vicious cycle where cytokine and ROS production are stimulated, both known to increase cardiovascular risk and tissue damage (Tecklenborg et al., 2018). Disturbance of immune balance in patients with kidney disease results in a continuous recruitment of immune cells and worsening of renal structure and function (Tecklenborg et al., 2018). The uremic toxins particularly have a pro-apoptotic and/or inhibitory effect on immune cells, contributing to the increased risk of infections observed in CKD patients. Among the main uremic toxins related to the activation of the immune system are: IS, PCS, *p*-cresyl glucuronide, β2-microglobulin, FGF-23, IL-6, and TNF-α among others (Fujii et al., 2018; Cohen, 2020).

Toll-like receptors (TLRs) are responsible for generating chronic inflammation observed in CKD once TLR4 being one of the key receptors involved in innate immunity and the recognition of LPS. Several UTs could potentially act as ligands in the activation of TLRs (Hauser et al., 2008). In this case, Sun et al. (2018) reported that UTs can serve as conditional pro-inflammatory DAMPs (danger signal-associated molecular patterns) or anti-inflammatory HAMPs (homeostasis-associated molecular patterns) and modulate inflammation. Consequently, classical DAMP receptors signaling activation such as TLRs, NLR-inflammasome-activated caspase-1 and other pro-inflammatory cytokines can elevate uremic toxins levels, as well as be inhibited by CD4^+^Foxp3^+^ regulatory T cells (Sun et al., 2018).

As reported in the literature, IS acts as a prooxidative and proinflammatory agent, triggers immune responses and stimulates CKD progression. The increase on plasmatic IS in chronic renal patients has been associated with changes in the hemostatic system, increased oxidative stress and monocyte activation (Wakamatsu et al., 2018). This molecule particularly shows a positive correlation with neopterin, a protein generated by macrophages and monocytes after stimulated by IFN-γ produced by activated T cells. As results, it’s possible to observe ROS production elevated and an increase on cellular adhesion molecules (CAMs) expression, supporting monocyte-endothelial cell interaction, leading to vascular inflammation and endothelial dysfunction. Thus, the direct role of IS in generating SRY-related HMG-box (SOX) and monocyte activation is demonstrated (Kaminski et al., 2017; Espi et al., 2020).

Girndt et al. (2020) reported that interactions between leucocyte and endothelial cells are promoted by IS through via p38 MAPK, a fundamental pathway triggering inflammatory cytokines such as IL-1β and TNF-α. Furthermore, the monocytes produce TNF-α after activation of AhR in response to IS. Also, the tumor necrosis factor stimulates the production of chemokine ligand CX3CR1L by endothelial cells, culminating with apoptosis (Girndt et al., 2020).

Another example of its effect on immune cells is that macrophage-like RAW 264.7 cells incubated with IS (as a prototype PBUT) exhibited increased NF-κB mRNA expression and decreased Nrf2 mRNA expression, promoting oxidative stress in these cells and triggering the secretion of TNF-α (Kim et al., 2017; Stockler-Pinto et al., 2018). Moreover, *in vivo* studies have shown that IS associated with another toxin, PCS or *p*-cresyl glucuronide plays pro-inflammatory effects, leading to vascular injury by stimulating the interaction between leukocytes and endothelium (Cohen, 2020). Both IS and PCS in high concentrations lead to an increase in MCP-1 (monocyte chemoattractant protein-1) expression, attracting macrophages and monocytes to the injured endothelium during the inflammatory process (Cohen, 2020). The uremic toxins are responsible for modulation of adhesion molecules such as P-selectin, E-selectin, ICAM-1 and vascular cell adhesion molecule-1 (VCAM-1), promoting the infiltration of macrophages and monocytes in endothelium (Da Cunha et al., 2020). In CKD patients, PCS can activate macrophages and also interferes in antigen processing impairing the adaptative immune response (Cohen, 2020).

On the other hand, in experiments using different PCS concentrations, showed an exacerbated immune response by macrophages demonstrated by an increase on NO production and by phagocytosis after early PCS accumulation. After aggravation of PCS levels during the disease evolution, immune cells became unable to respond to stimuli when a decrease on NO levels and phagocytosis ability are observed, configuring a immunosuppression state (Cohen, 2020). Moreover, PCS induced an increase in CD80 expression on the monocyte-derived macrophages (MDM) surface. However, patients with CKD have demonstrated an incorrect communication between cells involved in antigen presentation and T cells, since PCS was not able to induce high-level expression of HLA-DR and CD86 at the maximum dose (Azevedo et al., 2016).

Other studies show that PCS provokes activation of the TGF-β pathway with participation of klotho, a co-receptor for FGF-23, and through activation of the RAAS. This signaling stimulates leukocytes to produce ROS, which leads to renal tubular epithelial-to-mesenchymal transition, contributing to the progression of renal fibrosis (Fujii et al., 2018).

β2-microglobulin is also considered a uremic toxin with a high degree of toxicity. In contrast to the naive molecule, glycosylated β2-microglobulin has a pro-inflammatory effect on human monocytes, which can lead to lead to bone and joint degradation. Likewise, this protein has been associated with vascular stiffness, inflammation e bone remodeling parameters in several studies carried out with non-dialyzed and dialyzed CKD patients (Vanholder et al., 2018). Similar observations have been made for FGF-23, which has been involved in the decrease on neutrophil recruitment and host defense in experimental model of CKD. Besides, FGF-23 can immediately promote the release of inflammatory cytokines from liver, uncovering an interesting mechanism of chronic inflammation in CKD (Massy and Liabeuf, 2017). Lastly, we can mention inflammatory cytokines such as IL-6 and TNF-α. Both have a number of actions which are potentially deleterious in CKD patients because they promote inflammatory responses in different immune cells. Nonetheless, TNF-α is a weaker predictor of mortality than IL-6 (Castillo-Rodríguez et al., 2017).

**FIGURE 4:**
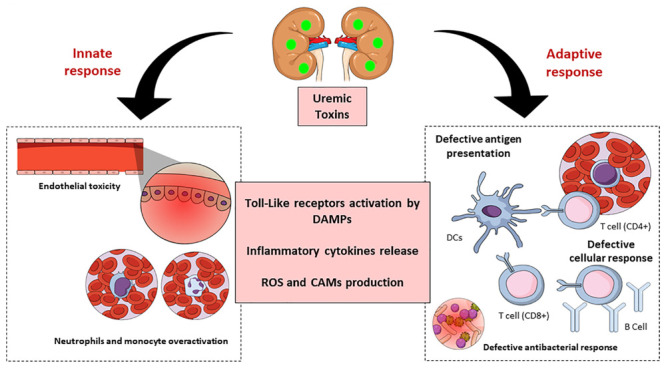
Schematic representation of chronic kidney disease (CKD)-associated immune dysfunctions. Chronic kidney disease leads to the accumulation of uremic toxins, which have an impact on innate and adaptive immune systems. The uremic toxins impair endothelial cells function and induce chronic low-grade activation of innate immune effectors (monocytes and neutrophils) through the participation of TLRs and inflammatory cytokines. These substances also affect the adaptive immune system causing a defective antigen presentation which brings with it a defective cellular and humoral response. DAMPS, damage-associated molecular patterns; ROS, reactive oxygen species; CAM, cellular adhesion molecules; DC, dendritic cells.

In summary, uremic toxins are key predictors of CKD (Figure 4). These substances affect the innate as well as the adaptive immune systems through many mechanisms, leading to the appearance of systemic pathologies in humans, and hence implicate the importance of their study, and advances in this field would greatly improve the clinical management of such patients.

## Clinical Approaches for Uremic Toxin Treatment

When focusing on protein-bound toxins, it is clear that they have an important role in patients who undergo dialysis, as the dialysis membrane does not filter them. Several studies have been focusing on this aspect in different approaches.

One possible alternative for filtering these toxins is the new types of dialysis which have been studied in the past years, such as high flux dialysis and others using different membranes. High flux membranes were designed to remove medium molecules, however, the clearance is not effective in molecules with molecular mass >15 kDa, such as myoglobulin (17 kDa), IL-6 (25 kDa), and FGF-23 (32 kDa) (Ronco, 2017; Wolley and Hutchison, 2018). A study by Sevinc et al. (2020) compared patients going thought high-flux hemodialysis followed by medium cut-off (MCO) membrane hemodialysis or vice versa, and the results showed that the levels of middle molecular mass molecules as myoglobulin and β2-microglobulin were reduced in the first and last sessions with MCO dialyzers compared to high-flux dialyzers, indicating that it is a possible way to lower the uremic toxin levels. Another important result in this study is that the albumin serum level decreased significantly after 3 months with MCO membrane (Sevinc et al., 2020). This result by itself is not enough to conclude whether the serum albumin level decrease is positive or not, but it is clear that more research should be conducted regarding it.

New strategies for performing dialysis have been used based on new nanomaterials. According to Ghosh and Roychaudhuri (2013) silicon nanoporous membranes were manufactured internally (SNMs), functionalized for effective elimination of UT. These membranes are about 15 nm with an average pore diameter of 8 nm, portable and more efficient. They have already been tested for urea and creatinine dialysis using custom Teflon devices of 2, 10, and 30 mL. It has been shown that the functionalization of the SNMs reduced the binding to proteins and the binding to the urea surface by 23% to insignificant values. The matrices eliminated about 42% of the urea and 48% of the creatinine from 30 mL of diluted serum samples in 15 min (Ghosh and Roychaudhuri, 2013; Ghosh et al., 2021).

Another study developed by Liu et al. (2021) showed that nitrogen-containing porous carbon adsorbent (NPCA) which are biosafe and efficient for the clearance of PBUTs were prepared from cross-linked acrylonitrile/divinylbenzene copolymer spheres followed by pyrolysis. The authors demonstrated that the effectiveness of PBUT removal was substantially greater than commercial adsorbents which are commonly used in clinical treatment of uremia (Liu et al., 2021). As mentioned before, AhR plays an important role in CRS, and thus, it must be taken as a possible target for clinical approaches. According to Miao et al. (2020), one way to inhibit AhR activation is through treatment with flavonoids. A treatment with 1-aminopyrene was able to mediate the AhR expression in both mice and cells, and also up-regulate its mRNA expression. These findings show that AhR could be activated by aryl-containing metabolites, which can be retarded by lowering the aryl-containing metabolite levels using dietary flavonoids. Flavonoids also seem to be able to attenuate renal fibrosis and down-regulate the mRNA expression of AhR target genes (Miao et al., 2020; Mo et al., 2020).

A study performed with knockout mice demonstrated that the animals AhR^–/–^ had moderate renal insufficiency compared to the wild type genes in males, while the difference was even lower in females. This could be explained by the lower level of basal expression of the hepatic enzymes such as p450 cytochrome CYP2E1 and the sulfotransferase SULT1A1 involved in IS production, suggesting that AhR^–/–^ mice have a weak capacity to metabolize indole into IS (Makhloufi et al., 2020).

Another possible target for therapeutic matters is the oral spherical carbon adsorbent AST-120. This substance is capable of reducing indole absorption through gastrointestinal sequestration. It is known that although AST-120 does not improve survival in CDK patients (Hatakeyama et al., 2012), it reduces their IS plasma levels (Pellicena and Schulman, 2014); however, there is no evidence of improving cardiovascular outcomes (Lano et al., 2020).

Other target when thinking about endogenous pathways to reduce toxin damage is the Klotho protein. This protein is expressed in many tissues, but it is more prominent in the kidneys (Kuro-o et al., 1999). Experiments *in vivo* have shown that Klotho gene transfer was able to reduce apoptosis after ischemic lesion and improve renal function (Kim et al., 2013). Regarding uremic toxins, it is known that IS and *p*-cresyl could inhibit Klotho gene expression in renal tubular epithelial cells (Sun et al., 2012a) while Klotho has the ability to counteract IS-induced cardiomyocyte hypertrophy by inhibiting oxidative stress and lessen the activation of p38 and ERK1/2 pathways (Yang K. et al., 2015), showing that Klotho is a promising target for therapeutical approaches.

## Final Considerations

Strong evidence suggests that uremic toxins are an alarming danger for cardiovascular system. Over the past 5 years there have been more than 400 publications about these molecules, and much remains to be discovered. The crosstalk between heart and kidney is close and undoubtedly involves molecules that are still unknown and mechanisms that have not yet been identified. Taken together, the study of the cellular and molecular effects of uremic toxins may elucidate new biomarkers and therapeutic targets in CKD. Uremic toxins are still a challenge to be explored.

## Author Contributions

CF, CJ, and AS: conceptualization, and writing – review and editing. FF-R, IV, and RC: writing – review and editing. MC-R: supervision, conceptualization, and writing – review and editing. All authors contributed to the article and approved the submitted version.

## Conflict of Interest

The authors declare that the research was conducted in the absence of any commercial or financial relationships that could be construed as a potential conflict of interest.

## Footnotes

1 http://uremic-toxins.org

## Abbreviations

α-SMA: alfa-smooth muscle actin
ADMA: asymmetric dimethylarginine
AGEs: advanced glycation end products
AhR: aryl hydrocarbon receptor
AKI: acute kidney injury
ARNT: aryl hydrocarbon receptor nuclear translocator
B2M: β _2_-microglobulin
BMP-2: bone morphogenetic protein
BTP: β-trace protein
CKD: chronic kidney disease
COX-2: cyclooxygenase-2
CRF: chronic renal failure
CRS: cardiorenal syndrome
CVD: cardiovascular diseases
CV: cardiovascular
DAMPs: danger signal-associated molecular patterns
DRE: dioxin response element
eGRF: epidermal growth factor receptor
eNOS: endothelial nitric oxide synthase
ER: endoplasmatic reticulum
Eutox: European Working Group on Uremic Toxins
FGF-23: fibroblast growth factor 23
GRF: glomerular filtration rate
IAA: indole-3-acetic acid
ICAM-1: intercellular adhesion molecule-1
IL6: Interleukin-6
IL1- β: interleukin 1 beta
IS: indoxyl sulfate
KLF2: Krüppel-like factor 2
KLF4: Krüppel-like factor 4
HAMPs: homeostasis-associated molecular patterns
HSA: human serum albumin
HSP 90: heat shock protein 90
HUVECs: human umbilical veins endothelial cells
HVSMCs: human vascular smooth muscle cells
LPS: lipopolysaccharides
LV: left ventricle
MAPK: mitogen-activated protein kinase
MBD: mineral bone disorder
MCP-1: monocyte chemoattractant protein-1
NADPH: nicotinamide adenine dinucleotide phosphate
NPCA: nitrogen-containing poros carbon adsorbent
NF- κ B: nuclear factor kappa-light-chain-enhancer of activated B cells
NLRP3: nucleotide-binding oligomerization domain leucine-rich repeat proteins 3
NO: nitric oxide
OATs: organic anion transporters
OPN: osteopontina
PAI-1: plasminogen activator inhibitor-1
PBUT: protein-bound uremic toxins
PCS: *p*-cresyl sulfate
PECAM-1: platelet endothelial cell adhesion molecule-1
Pi: inorganic phosphorus
Pit-: phosphate inorganic transporter 1
pRb: retinoblastoma protein
PTH: parathyroid hormone
RAAS: renin-angiotensin -aldosterone system
RAGE: receptor for advanced glycation end products
ROS: reactive oxygen species
SMAD 3: similar gene mothers against decapentaplegic 3
SNMs: silicon nanoporous membranes were manufactured internally
SOCS: suppressor cytokine signaling
SOX: SRY-related HMG-box
TF: tissue factor
TLRs: toll-like receptors
TIMP-1: tissue inhibitor of metalloproteinase 1
TMAO: trimethylamine-*N*-oxide
TGF- β 1: transforming growth fator β 1
TNF- α: tumor necrosis factor alpha
t-PA: tissue plasminogen activator
UPLC: ultra-performance liquid chromatography
VCAM-1: vascular cell adhesion molecule-1
VE-cadherin: vascular endothelial-cadherin
Vitamin D: 1,25-dihydroxyvitamin D (1,25[OH]_2_D_3_)]
VSMC: vascular smooth muscle cells
vWF: von Willebrand factor
ZO-1: zonula occludens-1.
